# Superior Efficacy of a Human Immunodeficiency Virus Vaccine Combined with Antiretroviral Prevention in Simian-Human Immunodeficiency Virus-Challenged Nonhuman Primates

**DOI:** 10.1128/JVI.00230-16

**Published:** 2016-05-12

**Authors:** Roger Le Grand, Nathalie Dereuddre-Bosquet, Stefania Dispinseri, Leslie Gosse, Delphine Desjardins, Xiaoying Shen, Monica Tolazzi, Christina Ochsenbauer, Hela Saidi, Georgia Tomaras, Mélanie Prague, Susan W. Barnett, Rodolphe Thiebaut, Alethea Cope, Gabriella Scarlatti, Robin J. Shattock

**Affiliations:** a Université Paris Sud, INSERM, CEA, DRF-Department of Immunology of Viral Infections and Autoimmune Diseases (IMVA), UMR1184, IDMIT Infrastructure, iMETI, Fontenay-aux-Roses, France; b Viral Evolution and Transmission Unit, Division of Immunology, Transplantation and Infectious Diseases, IRCCS San Raffaele Scientific Institute, Milan, Italy; c Mucosal Infection and Immunity Group, Department of Medicine, St. Mary's Campus, Imperial College London, London, United Kingdom; d Duke Human Vaccine Institute, Duke University, Durham, North Carolina, USA; e Department of Medicine and CFAR, University of Alabama at Birmingham, Birmingham, Alabama, USA; f INSERM U1219, INRIA SISTM, University of Bordeaux, Bordeaux, France; g Novartis Vaccines, Cambridge, Massachusetts, USA; h Vaccine Research Institute, Creteil, France

## Abstract

Although vaccines and antiretroviral (ARV) prevention have demonstrated partial success against human immunodeficiency virus (HIV) infection in clinical trials, their combined introduction could provide more potent protection. Furthermore, combination approaches could ameliorate the potential increased risk of infection following vaccination in the absence of protective immunity. We used a nonhuman primate model to determine potential interactions of combining a partially effective ARV microbicide with an envelope-based vaccine. The vaccine alone provided no protection from infection following 12 consecutive low-dose intravaginal challenges with simian-HIV strain SF162P3, with more animals infected compared to naive controls. The microbicide alone provided a 68% reduction in the risk of infection relative to that of the vaccine group and a 45% reduction relative to that of naive controls. The vaccine-microbicide combination provided an 88% reduction in the per-exposure risk of infection relative to the vaccine alone and a 79% reduction relative to that of the controls. Protected animals in the vaccine-microbicide group were challenged a further 12 times in the absence of microbicide and demonstrated a 98% reduction in the risk of infection. A total risk reduction of 91% was observed in this group over 24 exposures (*P* = 0.004). These important findings suggest that combined implementation of new biomedical prevention strategies may provide significant gains in HIV prevention.

**IMPORTANCE** There is a pressing need to maximize the impact of new biomedical prevention tools in the face of the 2 million HIV infections that occur each year. Combined implementation of complementary biomedical approaches could create additive or synergistic effects that drive improved reduction of HIV incidence. Therefore, we assessed a combination of an untested vaccine with an ARV-based microbicide in a nonhuman primate vaginal challenge model. The vaccine alone provided no protection (and may have increased susceptibility to a simian-HIV vaginal challenge), while the microbicide reduced the infection risk compared to that of vaccinated and naive animals. Importantly, the combined interventions provided the greatest level of protection, which was sustained following withdrawal of the microbicide. The data suggest that provision of ARV prophylaxis during vaccination reduces the potential for unexpected increased risks of infection following immunization and augments vaccine efficacy. These findings are important for the potential adoption of ARV prophylaxis as the baseline intervention for future HIV/AIDS vaccines.

## INTRODUCTION

The Thai RV144 vaccine trial, based on a canarypox virus vector prime-protein boost, is the first clinical trial to have shown moderate efficacy (31.2%) in cohorts at low risk of human immunodeficiency virus (HIV) exposure (1). Partial protection has also been achieved by other new biomedical approaches, including the use of antiretroviral (ARV) drugs for oral (44 to 75%) or topical (vaginal, 39%) preexposure prophylaxis (PrEP) (2–5). However, effectiveness was dependent upon consistent product use and impacted by multiple factors influencing susceptibility and exposure risk (6). Three decades of research on combined implementation of structural and behavioral interventions have indicated that combination approaches are more effective than any single intervention alone (7). Additional potential gains could be realized by assessing the impact of combining new biomedical prevention strategies (8). Indeed, a positive impact would be seen if combining ARV prevention and vaccines provided better protection than either intervention alone. Here, a reduction of the number of transmitted strains and/or a delay in the initial viral expansion phase might buy time for more effective immune clearance. Conversely, systemic immunity might curtail the dissemination of virus that bypasses the activity of topically applied ARVs. Furthermore, protection from productive infection on repeat exposure to HIV when using ARV prevention might evoke exposure-induced immunity. This could serve to modify vaccine-induced immune responses to better recognize a prevalent circulating virus. Indeed, evidence from some nonhuman primate (NHP) studies indicates that animals exposed to infectious virus when protected by PrEP demonstrate cellular immune responses to the challenge virus (9, 10). However, such immune responses in these nonvaccinated animals appeared insufficient to protect animals from a subsequent challenge in the absence of PrEP (10). Conversely, combinations could also have potential negative interactions. Certain vaccine-induced immune activation may have the potential to increase mucosal HIV-1 susceptibility (11, 12) that, in combination, could reduce the efficacy of ARV prevention. This has important implications, given that increased sensitivity over the potential of novel vaccines to enhance the risk of HIV acquisition may drive the adoption of oral PrEP provision as the baseline intervention for future HIV/AIDS vaccine trials.

## MATERIALS AND METHODS

### Ethics statement.

All 50 Mauritius-origin, outbred, young adult (4 to 6 years old), female cynomolgus monkeys (Macaca fascicularis) used were housed in the Commissariat a l'Energie Atomique (CEA) facilities (Fontenay-aux-Roses, France; CEA accreditation no. B 92-032-02) in compliance with the National Institutes of Health (NIH) Office of Laboratory Animal Welfare Guide for the Humane Care and Use of Laboratory Animals. This study and the procedures used were approved by the Comité Régional d'Ethique pour l'Expérimentation Animale Ile-de-France Sud (notification no. 10-062). All experimental procedures were carried out in the CEA animal facility in strict compliance with European guidelines for NHP care (European Directive 86/609 then; as of January 2013, EU Directive N 2010/63/EU) for protection of animals used for experimentation and other scientific purposes and the Weatherall report. Monitoring of the animals was under the supervision of the veterinarians in charge of the animal facilities. All efforts were made to minimize suffering, including improved housing conditions with enrichment opportunities (e.g., 12-h light–12-h dark scheduling, provision of treats such as biscuits supplemented with fresh fruit, and constant access to a water supply, in addition to regular play interaction with staff caregivers and research staff). Experimental procedures were performed while animals were under anesthesia with ketamine at 10 mg/kg (body weight). Euthanasia was performed prior to the development of symptoms of disease (indicated by a rapid decline in CD4^+^ T cells and/or an increase in viremia) and was performed by the intravenous injection of a lethal dose of pentobarbital. All 50 animals described were experimentally naive at the beginning of the study. Investigators were blind to the group allocations while performing immunological and virological assessments.

### Cynomolgus macaque combined microbicide-vaccine study.

Tenofovir gel (1%) or a control placebo gel, a proprietary formulation containing purified water with EDTA, citric acid, glycerin, methylparaben, propylparaben, and hydroxyethylcellulose (pH 4.5) provided by CONRAD (Arlington, VA), was transferred to 5-ml syringes and administered in 2-ml volumes via a 10FG soft catheter introduced ∼2 cm into the vagina. The process was atraumatic with no obvious leakage and was carried out while the animals were under anesthesia. Vaccine antigens, uncleaved gp140 TV1 and SF162, and MF59 adjuvant were provided and manufactured by Novartis. For each intranasal (i.n.) immunization, 50 μg each of TV-1 and SF162 gp140 was given in solution in a volume of 0.2 ml containing 500 μg of Resiquamod (R848) a TLR7/8 agonist (InvivoGen). The solution was dropped into each of the anterior nares of sedated animals placed in a prone position with their heads tilted back. For intramuscular (i.m.) immunizations, 100 μg each of TV-1 and SF162 gp140 was mixed with MF59 adjuvant and given in a volume of 0.4 ml into the deltoid muscle of the upper arm. Vaccinated cynomolgus macaques received three i.n. priming immunizations (0, 4, and 8 weeks), followed by two i.m. boost immunizations (16 and 28 weeks). Challenge studies were commenced 11 weeks after the final boost immunization.

Fifty Mauritius-origin, outbred, young adult (4 to 6 years old), female cynomolgus monkeys (M. fascicularis) were used for this study. No randomization was used; however, groups were balanced for susceptible and resistant major histocompatibility complex (MHC) haplotypes (H2, H6, and H4) (13). Recent studies demonstrated that the TRIM5α genotype has no impact on virus acquisition or vaccination outcome (14). Sample size was chosen knowing that 90% infection was expected in the control group on the basis of previous titration of the challenge stock. A sample size of 16 for each of the microbicide (M) and vaccine-plus-microbicide (V+M) groups was chosen with an 80% power to detect an increase in the survival proportion of 0.53 with a significance level (α) of 0.05 (two-tailed log rank test). A sample size of 12 animals for the controls provided an 80% power to detect an efficacy of 67% in the other groups.

**FIG 1:**
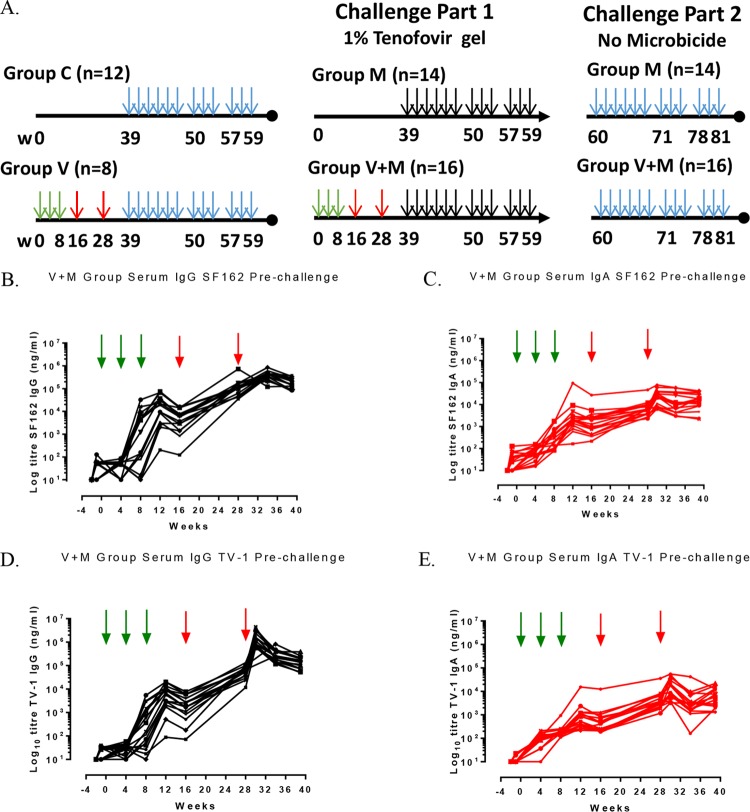
Vaccination-and-challenge schedules with longitudinal serum antibody responses in the V+M group prechallenge. (A) Schematic representation of vaccination-and-challenge schedules. Groups: V, vaccine alone; V+M, vaccine plus microbicide; M, microbicide alone; C, control. Green vertical arrows indicate i.n. vaccinations with R848 at weeks 0, 4 and 8; red vertical arrows indicate i.m. vaccinations with MF59 at weeks (w) 16 and 28; blue vertical arrows indicate 12 challenges in the absence of microbicide (weeks 39 to 59 and 60 to 80); and black vertical arrows indicate 12 challenges in the presence of microbicide (weeks 39 to 60). (B to E) SF162 serum IgG responses (B), SF162 serum IgA responses (C), TV-1 serum IgG responses (D), and serum IgA TV-1 responses (E) in the V+M group, all prechallenge.

Only eight animals were available for inclusion in the vaccine (V) group; however, this number was estimated to be sufficient to prove that the vaccine alone was ineffective at preventing infection. Investigators performing the animal studies were not blind to the group allocation. On the day of a challenge, ∼2 ml of the microbicide gel was applied atraumatically to the vagina (M and V+M groups) 1 h before a viral challenge. SHIV_162P3_ was added in a 1-ml volume containing 0.5 50% animal infective dose of an *in vivo*-titrated stock of the R5 virus SHIV_162P3_ (15), derived from the HIV-1 SF162 primary isolate and propagated in phytohemagglutin-activated rhesus macaque peripheral blood mononuclear cells (PBMC). Stock was obtained through the AIDS Research and Reference Reagent Program, Division of AIDS, National Institute of Allergy and Infectious Diseases, NIH (catalog no. 6526) (contributors: Janet Harouse, Cecilia Cheng-Mayer, and Ranajit Pal). Monkeys were bled weekly for viral load determination, and infection status was determined by measuring plasma viral loads with a reverse transcription (RT)-PCR assay with a sensitivity limit of 60 RNA copies/ml and a quantification limit of 300 RNA copies/ml (16). No further vaginal treatments were performed after the detection of viremia. Animals were monitored to determine set point viral loads. We excluded macaque 25015 from the M group because, at autopsy 2 months postinfection, we found a malformation of the genital tract, i.e., direct connection of the vagina, uterus, and peritoneal cavity. We also excluded animal 28413 from the M group because of a technical failure to deliver the full dose in a viral challenge. A schematic representation of the vaccination schedule, vaccination groups, and immunizations in parts 1 and 2 is shown in Fig. 1A.

### Immunogenicity antibody analysis.

TV-1- and SF162-specific binding antibodies were analyzed in serum and mucosal secretions. Briefly, 96-well plates were coated with a 1:1 ratio of anti-human κ and λ antibodies (Southern Biotech) to capture the standard curves (IgG or IgA standards) and TV1 and SF162 protein (1 μg/ml) to capture antigen-specific antibodies. Negative controls consisted of normal cynomolgus macaque serum and assay buffer. Standard curves of IgG and IgA consisted of 5-fold serial dilutions of purified IgG and IgA (starting at 1 μg/ml), and macaque serum and mucosal secretion samples were “screened” at 1:100 and 1:10, respectively, with samples and controls added in triplicate. Bound IgG was detected with goat anti-monkey IgG (Fc-specific) horseradish peroxidase (HRP) conjugate (Serotec), and bound IgA was detected with goat anti-monkey IgA (α-chain-specific) HRP conjugate (Autogen Bioclear). Following secondary antibody addition and development, plates were read at 450 nm. Positive responses were determined according to predefined cutoff values. Positive samples were titrated, and concentrations were determined by extrapolation of unknown samples against standards and expressed in micrograms of specific IgG or IgA per milliliter.

### Peptide array serum specificity mapping.

Serum epitope mapping of heterologous strains was performed essentially as previously described (17, 18). Briefly, a peptide library of overlapping peptides (15-mers overlapping by 12 amino acids), covering seven full-length HIV-1 gp160 Env consensus sequences (clades A to D; groups M, CRF1, and CRF2) and six vaccine and laboratory strain gp120 sequences (A244_1, TH023_1, MN_B, 1086_C, TV1_C, and ZM651_C) was printed onto epoxy glass slides (provided by JPT Peptide Technologies GmbH, Berlin, Germany). Microarray binding was performed with the HS4800 Pro Hybridization Station (Tecan, Männedorf, Switzerland). All arrays were blocked with Superblock T20 phosphate-buffered saline (PBS) blocking buffer for 0.5 h at 30°C, followed by a 2-h incubation at 30°C with heat-inactivated plasma diluted 1:250 in Superblock T20. Arrays were incubated for 45 min at 30°C with goat anti-human IgG conjugated with DyLight649 (1.5-μg/ml final concentration; catalog no. 109-495-098; Jackson ImmunoResearch, West Grove, PA) diluted with Superblock T20. Washing between steps was done with PBS containing 0.1% Tween. Arrays were scanned at a wavelength of 635 nm with an Axon GenePix 4300 Scanner (Molecular Devices, Sunnyvale, CA) at a photomultiplier setting of 540 and 100% laser power. Images were analyzed with GenePix Pro 7 software (Molecular Devices). The intensity of postimmunization serum binding to each peptide was corrected with its own background value, which was defined as the median signal intensity of the prebleed serum for that peptide plus 3 times the standard error of the three subarray replicates present on each slide.

### Neutralization TZM-bl assay.

Viral titration and neutralization assays were performed as previously described (19, 20). Pseudotyped viruses (PSVs) pCAGGSSF162gp160, BX08, 93MW965.26, TV1.21, TV1.29, QH0692, DJ263.8, and pSV7d-SHIVSF162-Qlc32 4014; the infectious molecular clone (IMC) pNL-LucR.T2A-SHIV162P3.5.ecto; and the culture supernatant of SHIV162P3 M623-Derived were used. The JC53bl-13 (TZM-bl) cell line (catalog no. 5011) was obtained from the National Institute for Biological Standards and Control (NIBSC) Center for AIDS Reagents, United Kingdom, and validated as mycoplasma free with MycoAlert (Lonza). Four 3-fold dilutions, starting with 1/20 of each serum or mucosal sample, were incubated with virus supernatant (200 tissue culture infective doses) for 1 h. Thereafter, 10^4^ TZM-bl cells were added, the plates were incubated for 48 h, and then luciferase activity was measured. The positive controls were sera of HIV-1-infected individuals or macaques and monoclonal antibodies known to neutralize the viruses. The neutralization titer was defined as the sample dilution at which the number of relative luminescence units (RLU) was 50% of the number of RLU in virus control wells after subtraction of the background number of RLU in control wells with only cells.

### ADCC activity.

Antibody-dependent cell-mediated cytotoxicity (ADCC) was tested as previously described (21), with the IMC pNL-LucR.T2A-SHIV162P3.5.ecto. IMC-infected CEM.NKR.CCR5 cells (obtained from the NIBSC Center for AIDS Reagents, United Kingdom [catalog no. 0099], and validated as mycoplasma free with MycoAlert [Lonza]) were incubated at a 1:30 ratio with PBMC and six 4-fold dilutions of each serum starting with a 1:100 dilution. The percentages of cells positive for the GzB substrate are reported as percentages of granzyme B activity. The positive control was monoclonal antibody b12.

### IFN-γ and IL-2 T-cell ELISpot assay.

Enzyme-linked immunosorbent spot (ELISpot) assays were performed with MultiScreen 96-well filtration plates (Millipore, Guyancourt, France) coated overnight at 4°C with a monoclonal antibody against monkey gamma interferon (IFN-γ; clone GZ-4; Mabtech, Nacka, Sweden) and interleukin-2 (IL-2; CT-611 kit; U-Cytech Biosciences, Utrecht, The Netherlands) in accordance with the manufacturer's instructions. The plates were washed five times with PBS and then blocked by incubation for 1 h at 37°C with RPMI 1640 medium containing glutamax-1 (Gibco, Life Technologies, United Kingdom) supplemented with 10% heat-inactivated fetal calf serum (FCS; Lonza) (culture medium). Freshly isolated PBMC (2 × 10^5^ per well) were stimulated in duplicate at 2 μg/ml with an HIV-1 gp120 SF162 recombinant protein (batch MID167d; Novartis) or with SIVmac251 Gag peptide pools (15-mers overlapping by 11 amino acids). Control wells (10^3^ PBMC) were stimulated with medium alone or with a phorbol myristate acetate (1 μg/ml). The plates were incubated for 24 h (gp120 glycoprotein) or 18 h (Gag peptide pools) at 37°C in a 5% CO_2_ atmosphere. They were then washed five times with PBS. A biotinylated anti-IFN-γ (clone 7-B6-1; Mabtech) or anti-IL-2 (CT-611 kit; U-Cytech Biosciences) antibody was then added at a concentration of 1 μg/ml in 0.5% FCS in PBS, and the plates were incubated overnight at 4°C. The plates were then washed five times with PBS, incubated with 0.25 μg/ml alkaline phosphatase-streptavidin conjugate (Sigma-Aldrich, St-Quentin Fallavier, France) for 1 h at 37°C, and washed five times with PBS. Spots were developed by adding nitroblue tetrazolium–5-bromo-4-chloro-3-indolylphosphate substrate (Sigma-Aldrich) and counted with an automated ELISpot assay reader (ELR04 XL; Autoimmun Diagnostika GmbH, Strassberg, Germany).

### Statistical analyses.

A time-to-event analysis was conducted with first Kaplan-Meier estimates by using log rank tests comparing groups for data sets in phase A and data sets in phase B. We then used a logistic model with random effects taking into account repeated measurements of each macaque. This type of model allows the discrete exposure to the infection due to the challenges at given times to be taken into account. Preliminary analyses showed that the variance of the random effect was not significantly different from zero (*P* = 0.25), indicating little response variability between monkeys. The first analysis of interest was logit(*P*_infection_ = 1) = α0 + α1*t* + α2*G*, where *t* represents the time since vaccination and *G* represents the treatment group. The time of infection was taken as the date of the previous challenge prior to the first SIV-positive test result. However, sensitivity analysis, taking the time of infection as the previous challenge or before the previous challenge, did not change the results qualitatively (results not shown). The rationale of including *t* in the regression is to take into account any residual confounding associated with a change in the probability of infection over time. This could be due to the selection of the population, those being the most resistant being uninfected until the end. This analysis was run on the data set of parts 1 and 2. We then extended the analysis by dissociating the effects of vaccination (*V*) and microbicide (*M*) and their interaction (*V* · *M*) as follows: logit(*P*_infection_ = 1) = α0 + α1*t* + α2*V* + α4*V* · *M*. Second-order interactions among *M*, *V*, and *t* were tested but were not significant (*P* = 0.91). However, in the final analysis, we used logit(*P*_infection_ = 1) = α0 + α1*t*_*p*2_ + α2*V* + α3*M* + α4*V* · *t*_*p*2_ + α5*V* · *M*. Time (*t*) was categorized as an indicator of part 1 or 2, *t*_*p*2_ = *I* (*t* = >22weeks), and the effect of the interaction *V* · *t*_*p*2_ was kept (*P* = 0.22). Results were used to compare the effects of *V*+*M* in parts 1 and 2. This analysis was run on the pooled data set of parts 1 and 2. All results are reported as odds ratios (ORs) together with their 95% confidence intervals (CIs) and *P* values for statistical significance. Results are presented as percentages of risk reduction, but it should be kept in mind that ORs are only approximations of risk estimates. We compared the results of logistic regression with the time-to-event Cox model analysis results. Hazard ratios and ORs give similar conclusions (results not shown). Analyses were run with R software and the packages “survival” for survival and “lme4” for logistic mixed-effect models.

## RESULTS

### Study design.

In this study, we use an NHP model to determine potential interactions of combining a microbicide with an envelope-based vaccine over either intervention alone. We chose to study vaginal transmission, as most vaccine and microbicide efficacy trials will likely be dependent upon the use of trial sites in sub-Saharan Africa, where the infection rates are highest among women (22). We chose to evaluate a 1% tenofovir microbicide gel on the basis of its reported efficacy in the CAPRISA 004 trial (5). We focused on an HIV-1 envelope-based vaccine, reflecting the likely protective role of antibody in RV144, and adopted a vaccine strategy previously shown to protect NHPs against a vaginal challenge with tier 1 SHIV_SF162p4_ (23). We designed a two-part study to test the potential interactions (positive or negative) between these two biomedical strategies over single interventions. In part 1, we compared the protective efficacies of the envelope-based vaccine (V group), 1% microbicide tenofovir gel alone (M group), and their combination (V+M group) against 12 repeat vaginal challenges with the tier 2 SHIV strain SF162P3 (SHIV_SF162p3_) (Fig. 1A). Critical to the experimental design was that neither of the individual interventions could be fully protective by itself; therefore, we elected to use SHIV_SF162p3_ rather than more closely matched strain SHIV_SF162p4_, against which vaccination was previously shown to provide 100% protection (23). In part 2, protected animals were challenged a further 12 times in the absence of microbicide.

### Vaccination and vaginal SHIV challenge.

**FIG 2:**
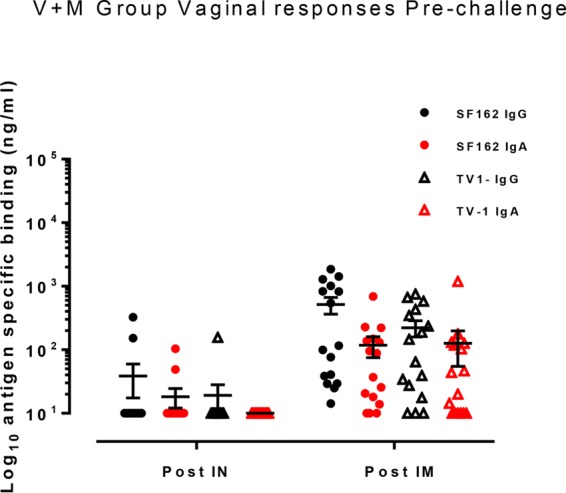
Vaginal binding antibody responses in the V+M group only postimmunization prior to a challenge. Antibody responses were measured in vaginal secretion samples collected after i.n. immunization (Post IN) at week 12 and after i.m. immunization (Post IM) at week 34 prior to a challenge. SF162- and TV-1-specific IgG antibodies are represented by black symbols, and red symbols show SF162- and TV-1-specific IgA responses. A short horizontal line with error bars indicates the mean ± the standard error of the mean of each group.

**FIG 3:**
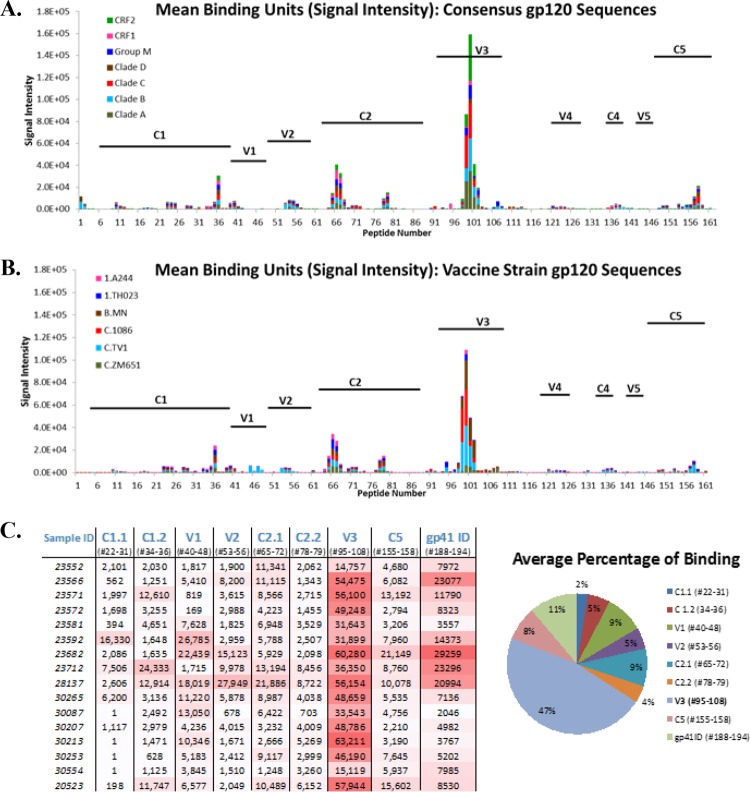
Mapping of serum IgG binding to gp120 linear epitopes by peptide microarray. (A, B) Representative plots of gp120 binding in the serum of an immunized animal binding to either consensus gp120 sequences or vaccine strain gp120 at the serum IgG response peak (2 weeks after the last immunization). The values on the *x* axis are peptide numbers in the array library. Those on the *y* axis are signal intensities (baseline subtracted). Differently colored bars represent the different strains/clades indicated. The variable (V) and constant (C) domains of gp120 are labeled in each panel. (C) Intensity of binding to each epitope identified in the animals. The definition of each epitope as the range of peptide numbers in the array library is listed under each epitope. Color coding highlights higher intensity in darker red. The pie chart shows the average binding to each epitope as a percentage of the total. Each pie slice represents the mean value for all of the animals of maximum binding to the specified epitope/sum of maximum binding of all of the epitopes.

**FIG 4:**
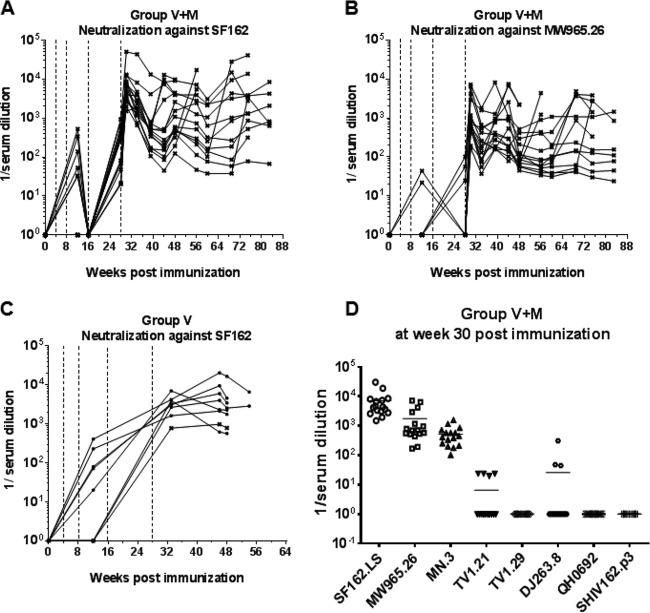
Neutralizing antibody titers of groups V+M and V. (A to C) Kinetics of neutralizing response after each immunization in the V+M (A, B) and the V (C) groups. Sera were tested against PSV SF162 or 93MW965.26 in a TZM-bl assay. Dotted lines represent vaccinations at weeks 0, 4, 8 (i.n. immunizations with R848), 16, and 28 (i.m. immunizations with MF59). (D) Neutralizing antibody titers of NHP in the V+M group 30 weeks after the first immunization against a panel of PSVs of tiers 1 and 2 in a TZM-bl assay. Solid horizontal lines in panel D indicate mean neutralizing titers.

**FIG 5:**
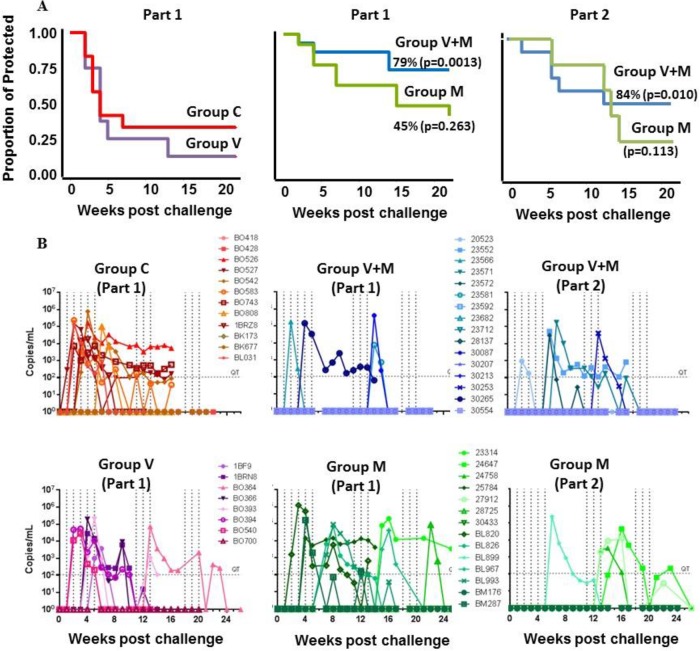
Time-to-event survival analysis Kaplan-Meier curves and infection of macaques determined by RT-PCR in plasma. Groups: V, vaccine alone; V+M, vaccine plus microbicide; M, microbicide alone; C, control. (A) Kaplan-Meier plots of animals confirmed infected by RT-PCR. Red, control animals; purple, V group; blue, V+M group; green, M group. (B) Plasma viral loads of individual macaques during challenge phases. Vertical dotted lines indicate the dates of challenges; horizontal lines indicate the limit of quantification (300 copies/ml).

Cynomolgus macaques in the vaccine groups (V and V+M groups) received three i.n. priming immunizations (0, 4, and 8 weeks) with a combination of two gp140 uncleaved trimers (TV-1 clade C plus SF162 clade B) coadministered with R848 (TLR7/8) adjuvant, followed by two i.m. boost immunizations (16 and 28 weeks) delivered with MF59 adjuvant. This induced robust serum binding antibody responses (IgG and IgA) to both immunogens (TV-1 and SF162 gp140) that remained stable through to week 39, the start of the challenge (Fig. 1B to E). Weak vaginal responses were observed in some animals after i.n. priming, and they were boosted following i.m. immunization (Fig. 2). Peptide array analysis demonstrated that all of the animals developed a strong cross-clade anti-V3 response and responses to the gp41 immunodominant region. Animals also developed cross-clade responses of lower intensity to V2, C2, and C5 gp120 epitopes (Fig. 3). Autologous serum neutralizing antibodies to HIV-1 SF162 were induced following i.m. boosts (7,891 ± 11,728 [mean ± standard deviation]) that decreased by approximately 1 log prior to a challenge (Fig. 4A). Neutralizing antibodies were absent from vaginal secretions or below the level of detection. Serum neutralizing antibody responses to clade C 93MW965.26 (1,757 ± 2,240) were also induced by week 30, but those to TV1 or tier 2 viruses of other subtypes were not (Fig. 4B and D). As similar responses had been fully protective against tier 1 SHIV_SF162p4_ (23), we elected to use the variant SHIV_SF162p3_, which differs by 22 amino acids and contains an additional glycan at the N-terminal base of the V2 loop predicted to confer escape from autologous neutralization (24). Prechallenge sera and vaginal samples were confirmed to have little or no neutralizing activity against SHIV_SF162p3_ (Fig. 4C). As predicted on the basis of neutralization, the vaccine alone (group V) did not protect against 12 consecutive low-dose intravaginal challenges with SHIV_SF162P3_ (Fig. 5A and Table 1), where 50% of the animals in both the V and naive control (C) groups became infected after two challenges (Table 2). Strikingly, there were more infections in the V group than in the C group, with an OR of 1.73, although this did not reach statistical significance (*P* = 0.341; Table 1), and the baseline risk of infection in the V group was almost double that in the C group (Table 2). Furthermore, the vaccine alone had no impact on viral load kinetics or control (Fig. 5B).

**TABLE 1:** OR for group effects on the probability of infection in part 1^*a*^

Group effect	OR	95% CI	*P* value
Intercept	0.22	0.09, 0.53	<0.001
Time	0.94	0.87, 1.00	0.077
V+M vs C	0.21	0.06, 0.71	0.013
M vs C	0.55	0.20, 1.56	0.263
V vs C	1.73	0.56, 5.32	0.341

a Reference: group C.

**TABLE 2:** Summary analysis of infection risk in part 1

Animal group	No. of protected animals/total	% of protected animals	No. of challenges	No. of challenges to infect 50% of animals (95% CI)	*P* value^*a*^	HR (95% CI)^*b*^	Baseline risk of infection
C	4/12	33	12	2.0 (1.0, undetermined)			0.087
V	1/8	13	12	2.0 (2.0, undetermined)	0.568	1.53 (0.55, 4.26)	0.159
M	6/14	43	12	9.5 (5.0, undetermined)	0.272	0.56 (0.21, 1.49)	0.068

a Versus group C (Mantel-Cox test) overall, *P* = 0.00.

b HR, hazard ratio; Cox model for time to infection.

### Tenofovir gel and vaginal SHIV challenge.

The microbicide regime was designed to be partially protective; in our study, 1% tenofovir gel was applied vaginally 1 h before each of 12 sequential vaginal challenges. We confirmed in cynomolgus macaques that tenofovir disoproxil fumarate (TDF) levels measured in peripheral blood and genital tissue at different time points following 1% tenofovir gel application reached concentrations compatible with local antiviral activity (Table 3). We also measured TDF diphosphate in genital tissues as a means to quantify the active drug phosphorylated by local exposed cells. In both cases, levels similar to those reported in rhesus macaques (25) and in humans in the CAPRISA 004 trial were observed (26). To limit the number of animals included in this study (50 in total), we assumed the risk of not detecting a benefit of the partially effective microbicide alone (group M) relative to naive animals (group C). This was indeed the case compared to naive controls (OR, 0.55; *P* = 0.263; Table 1). However, on the basis of the Kaplan-Meier curves (Fig. 5A), 9.5 challenges would be required to infect 50% of the animals in group M, compared to 2 challenges for those in groups C and V (Table 2). The microbicide alone provided a 68% reduction in the risk of infection relative to that of the vaccine group (OR, 0.32; *P* = 0.045; Table 4). There was, however, no apparent impact of tenofovir gel on viral load kinetics in animals that became infected (Fig. 5B).

**TABLE 3:** Pharmacokinetics of vaginally applied 1% tenofovir gel^*a*^

Drug and location	Mean *C*_max_^*b*^ (ng/g) ± SD	*T*_max_^*c*^ (h)	Mean AUC_0–12_^*d*^ (ng · h/g) ± SD
TDF			
Serum	349 ± 196	1	793 ± 388
Vagina	151,533 ± 89,312	1	877,034 ± 133,061
Exocervix	286,960 ± 169,805	1	756,546 ± 277,502
Endocervix	39,472 ± 29,670	1	98,139 ± 36,024
Uterus	14,449 ± 6,068	1	38,967 ± 13,847
TDF-DP			
Vagina	94 ± 4	4	668 ± 142
Exocervix	64 ± 16	4	883 ± 571
Endocervix	<10		
Uterus	<10		

a The pharmacokinetics of TDF and TDF diphosphate (TDF-DP) in female genital tract tissues and serum after intravaginal administration of 1% tenofovir gel were determined. Tissues were sampled at necropsy (two animals per time point), which was performed at 0, 1, 4, or 12 h after gel administration. Tenofovir was quantified in serum by high-performance liquid chromatography–tandem mass spectrometry. Lower limits of quantification: 10 ng/g in vaginal, exocervical, and uterine tissues and 25 ng/g in endocervical tissue. TDF-DP in serum was quantified by high-performance liquid chromatography–tandem mass spectrometry. The lower limit of quantification was 10 ng.

b *C*_max_, maximum concentration of drug in serum.

c *T*_max_, time to *C*_max_.

d AUC_0–12_, area under the concentration-time curve from 0 to 12 h.

**TABLE 4:** OR for group effects on the probability of infection in part 2^*a*^

Group effect	OR	95% CI	*P* value
Intercept	0.38	0.15, 0.95	0.039
Time	0.94	0.87, 1.01	0.077
V+M vs V	0.12	0.03, 0.44	0.001
M vs V	0.32	0.11, 0.98	0.045
C vs V	0.58	0.19, 1.78	0.341

a Reference: group V.

### Vaccine-microbicide combination provides enhanced protection.

As the primary objective was to assess the potential benefit of applying the vaginal microbicide approach to previously vaccinated animals, this study was designed to determine the efficacy of the combination (V+M group, *n* = 16) and that of either intervention alone. V+M group animals were vaccinated in parallel with group V, and challenge studies commenced at week 39. In these animals, the microbicide was applied in an identical fashion to that used for group M in each of the 12 challenges in part 1 (Fig. 1A). The V+M combination provided a 79% reduction in the per-exposure probability of infection (*P* = 0.013; Table 4) relative to that of naive controls, an 88% reduction (OR, 0.12; *P* = 0.001; Table 1) relative to vaccine alone and a 63% reduction (OR, 0.39) relative to microbicide alone, although this did not reach statistical significance (*P* = 0.114) (Fig. 5A). Only four animals were infected after 12 repetitive challenges, too few to predict the number of challenges needed to reach 50% infection in the V+M group. Animals that remained uninfected following 12 consecutive intravaginal challenges in the presence of microbicide (challenge part 1) immediately progressed to challenge part 2 (Fig. 1A). Here, all protected animals received a further 12 sequential challenges, irrespective of their initial assignment to the M (*n* = 6) or V+M (*n* = 12) group, to determine susceptibility in the absence of microbicide. By the end of part 2 (Fig. 5A) there was still no statistically significant difference between group M and untreated controls in part 1 (Table 5), while in the V+M group, there was an 84% reduction in the per-exposure probability of infection (*P* = 0.010) relative to that of untreated controls and an 86% reduction in the per-exposure probability of infection relative to that of vaccine alone (*P* = 0.002). Here, the positive interaction between the microbicide and the vaccine remained the same (interaction coefficient *P* = 0.13). In order to gain further insight, we discretized the time into parts 1 and 2 and reran the analysis. This analysis indicated that the microbicide alone over the entire course of parts 1 and 2 showed a trend toward protection (OR, 0.26 [0.06:1.02]; *P* = 0.054; Table 6), whereas the microbicide and vaccine combined provided significant protection, with a 91% reduction in the per-exposure probability of infection (*P* = 0.004; Table 6). Further analysis was performed to investigate the potential interaction between time and vaccination in the V+M group (discretized in parts 1 and 2). The effect of the V+M group compared to the control increased in part 2, providing a 98% (*P* = 0.002) reduction in the per-exposure probability of infection in part 2 compared to 89% (*P* = 0.010) in part 1, indicating a long-term effect of vaccination even without the microbicide (Table 7). We excluded potential confounders that might have influenced differences in susceptibility. The distribution of the MHC genotype was equal across the different groups (13). Furthermore, recent studies demonstrated that the TRIM5α genotype has very little variability in Mauritian cynomolgus macaques and has no impact on virus acquisition or vaccination outcome (14). To more faithfully replicate the human condition, animals were not treated with Depo-Provera, which is often used to enhance susceptibility to infection. All of the animals were naturally cycling. Analysis of progesterone levels showed no overrepresentation in any group of animals in the follicular phase, associated with heightened susceptibility.

**TABLE 5:** OR for treatment type on the probability of infection in parts 1 and 2 combined

Group effect	OR	95% CI	*P* value
Intercept	0.23	0.11, 0.47	<0.001
Time	0.95	0.92, 0.98	0.004
V vs C	1.17	0.49, 2.82	0.720
M vs C	0.46	0.18, 1.20	0.113
V+M vs C	0.16	0.04, 0.65	0.010
V+M vs V	0.14	0.04, 0.50	0.002

**TABLE 6:** OR for treatment type on the probability of infection in parts 1 and 2 combined^*a*^

Group effect	OR	95% CI	*P* value
Intercept	0.22	0.09, 0.57	0.001
Time in phase B	0.35	0.10, 1.19	0.093
V vs C	0.97	0.28, 3.28	0.958
M vs C	0.26	0.06, 1.02	0.054
V+M vs C	0.09	0.02, 0.46	0.004
V+M vs V	0.09	0.02, 0.45	0.003

a Discretized time.

**TABLE 7:** OR for treatment type on the probability of infection in parts 1 and 2 combined depending on time^*a*^

Group effect	OR	95% CI	*P* value
Intercept	0.17	0.06, 0.47	0.001
Time in phase B	0.75	0.14, 4.09	0.738
V vs C	1.91	0.39, 9.28	0.425
M vs C	0.37	0.09, 1.59	0.185
V+M in phase A vs C	0.11	0.02, 0.59	0.010
V+M in phase B vs C	0.02	0.001, 0.22	0.002

a Interaction of order 2 vaccine by microbicide by time.

### Immune parameters modulated by protected exposure to infectious SHIV.

**FIG 6:**
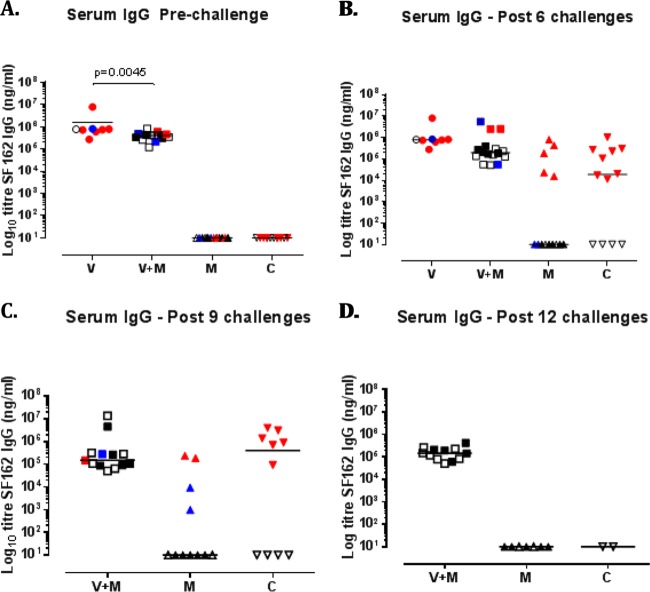
Vaccine-induced binding antibodies to SF162 in serum. Groups: V, vaccine alone; V+M, vaccine plus microbicide; M, microbicide alone; C, control. (A to D) Serum response antibody levels in nanograms per milliliter prechallenge at week 34 (A), after 6 challenges (B), after 9 challenges (C), and after 12 challenges (D). Colors are indicative of the time at which infection was detected by plasma viremia as follows: red, during the first 6 challenges (until week 9 postchallenge); blue, during challenges 7 to 12 (weeks 11 to 21 postchallenge); black, during challenges 13 to 24 (from week 22, in the absence of microbicide). Empty symbols represent animals that did not show signs of infection.

**FIG 7:**
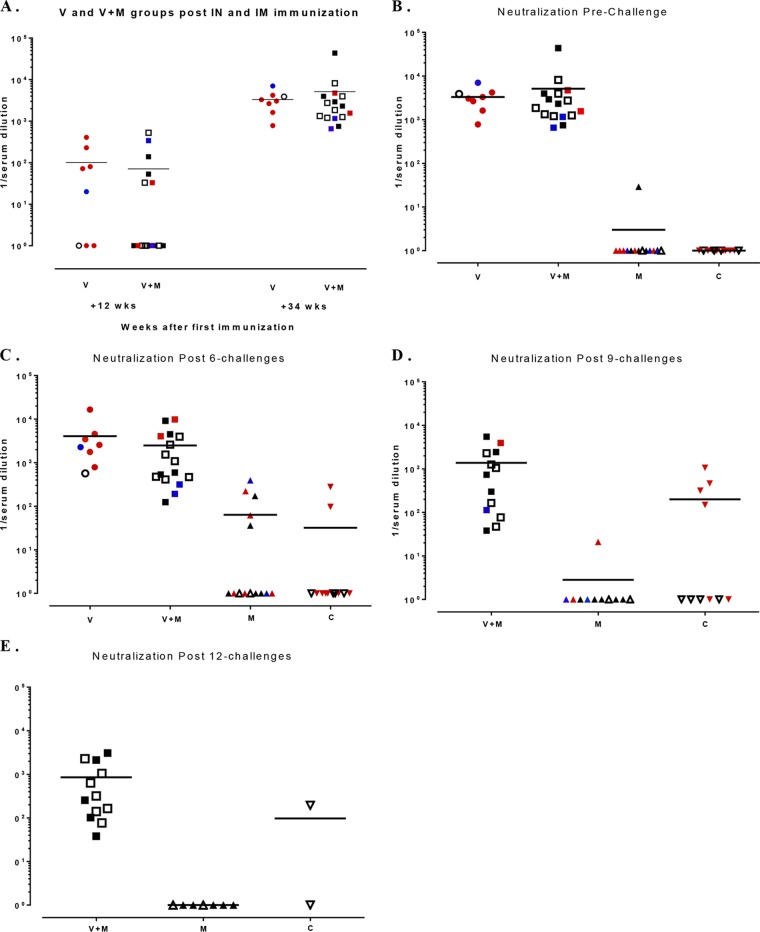
Titers of neutralizing antibodies to SF162 in serum. Sera were analyzed by TZM-bl assay at 12 weeks after i.n. immunization and at 34 weeks after i.m. immunization (A), prechallenge (B), after 6 challenges (C), after 9 challenges (D), and after 12 challenges (E). Each symbol represents one animal, and colors are indicative of the times when infection was detected by plasma viremia as follows: red, during the first 6 challenges (up to 9 weeks postchallenge); blue, challenges 7 to 12 (weeks 11 to 21 postchallenge); black, challenges 13 to 24. Empty symbols represent animals that were not infected. There was no statistically significant difference between the V and V+M groups at any point.

**FIG 8:**
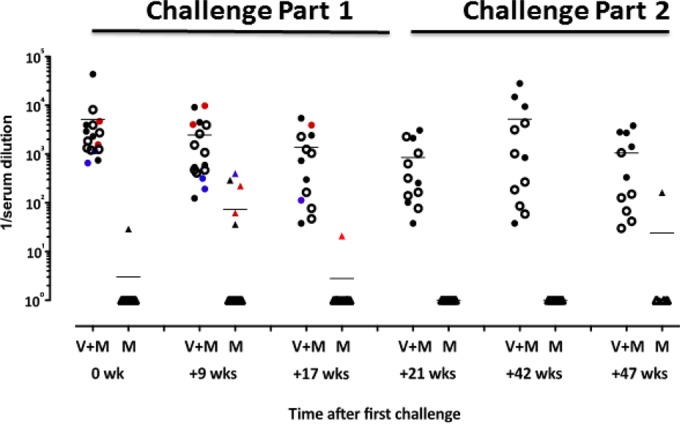
Neutralizing antibody responses to SF162 during challenge phase parts 1 and 2. TZM-bl assay neutralization data 5 weeks before a challenge (0 wk); 9, 17, and 21 weeks after the first challenge with microbicide; 1 week after the 12 challenges without microbicide (week 42); and 5 weeks thereafter (week 47). Each symbol represents one animal. Colors are indicative of the times when infection was detected by plasma viremia as follows: red, during the first 6 challenges (until week 9 postchallenge); blue, during challenges 7 to 12 (weeks 11 to 21 postchallenge); black, during challenges 13 to 24 (from week 22, in the absence of microbicide). Empty symbols represent animals that were not infected. The mean of each group is indicated by a solid line. For V+M versus V, *P* < 0.0001 (Mann-Whitney test).

Subsequently, we assessed potential immune parameters that might be associated with enhanced protection in the V+M group relative to that in the V or M group. Prior to a challenge, the serum or mucosal antibody titers in the V and V+M groups were similar although the serum antibody titers were slightly raised in the V-only group (*P* = 0.0045, Fig. 6A and 1B to D). Titers of induced serum binding antibodies to SF162 and TV-1 gp140 were high prechallenge and may have masked any potential boosting effects of protected exposure in the V+M group (Fig. 1B). However, there was no evidence of boosting in the mucosal samples of protected animals over time. There was little or no neutralization of SHIV_SF162P3_ in serum and mucosal samples prior to a challenge in both the V and V+M groups (Fig. 4) and no evidence of an induced response in protected animals at any point during parts 1 and 2. However, autologous neutralizing responses to HIV-1_SF162_ were equivalent in the V+M group relative to the V group prior to a challenge and postimmunization (Fig. 7A), with no evidence of boosting in protected animals after 6, 9, or 12 challenges in part 1 (Fig. 7B to E) or change in epitope recognition assessed by peptide array analysis (data not shown). In addition, there was no evidence of boosting of neutralizing responses to HIV-1_SF162_ or 93MW965.26 in protected animals in part 1 in the presence of microbicide or in part 2 in the absence of microbicide (Fig. 8). Furthermore, ADCC responses to SHIV_SF162P3_ were absent from all of the uninfected animals at all of the time points examined.

**FIG 9:**
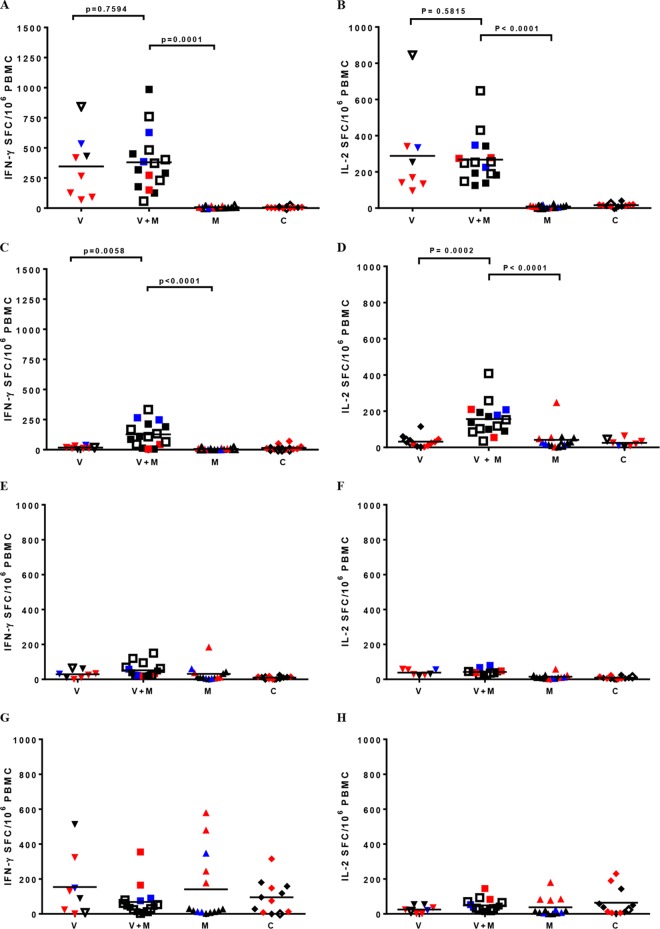
T-cell responses measured by ELISpot assay pre- and postchallenge. gp120-specific T-cell ELISpot assay responses in numbers of spot-forming cells/10^6^ PBMC prechallenge (A, B) and after six challenges (C, D) and SIV Gag-specific T-cell ELISpot assay responses prechallenge (E, F) and after six challenges (G, H) are shown. Red symbols indicate infection during the first six challenges, blue symbols indicate infection during challenges 7 to 12 (weeks 11 to 21 postchallenge), and black symbols indicate infection during challenges 13 to 24. Empty symbols represent animals that were not infected. Solid lines indicate mean values. *P* values, where relevant following statistical analysis, are also shown.

Robust cellular immune responses to SF162 gp120 were detected by ELISpot assay in the V and V+M groups after the five immunizations. Responses were similar in both groups Fig. 9, with mean numbers of spot-forming cells per million PBMC of 369 ± 246 and 274 ± 171 for IFN-γ and IL-2, respectively (Fig. 9A and B). There was no correlation between prechallenge vaccine-induced IFN-γ and IL-2 gp120-specific responses and protection observed after challenges in parts 1 and 2. We also measured T-cell responses to gp120 stimulation after the first sequence of challenges. These had significantly decreased in group V compared to prechallenge measurements in week 34 (*P* = 0.0058 and *P* = 0.0002 for IFN-γ and IL-2, respectively, Fig. 9C and D), down to the level of control animals (group C). The responses of repeatedly exposed nonvaccinated animals in group M were similar (*P* = 0.7315 and *P* = 0.9027 for IFN-γ and IL-2, respectively) to those of control animals (group C), indicating no added benefit for naive animals when they were exposed to the virus in the presence of microbicide.

Remarkably, the responses of vaccinated animals appeared to be significantly increased when they were exposed to SHIV_SF162P3_ following treatment with TDF gel (V+M group) in comparison to those of animals in groups V (*P* = 0.0058 and *P* = 0.0002 for IFN-γ and IL-2) and M (*P* < 0.0001 and *P* < 0.0001 for IFN-γ and IL-2), demonstrating that repeated challenges of preimmunized animals when using microbicides for prevention evoke exposure-induced immunity. However, responses raised in protected animals in this group were similar to those of nonprotected macaques. All of the animals infected in parts 1 and 2 demonstrated robust responses to SIV Gag peptide pools, irrespective of the intervention group (Fig. 9G and H); however, there were no detectable anti-Gag responses in any of the protected animals following sequential challenges in parts 1 and 2, irrespective of the intervention group (M or V+M).

## DISCUSSION

The primary aim of this study was to determine any potential superiority of a microbicide-vaccine combination to single prevention approaches. The lack of protection by the vaccine alone and lack of virologic control upon a challenge with the tier 2 autologous escape variant SHIV_SF162p3_ is unsurprising. This contrasts with the earlier observation of protection against a high-dose challenge with SF162 immunogen-matched tier 1 SHIV_SF162p4_ (23). These data reflect the dominant role of neutralizing antibodies in sterilizing protection, while the absence of induced ADCC activity likely accounts for the lack of impact on virologic control. However, the observation that 7/8 animals were infected in the vaccine group versus 4/12 in the control group, although not statistically significant, is troubling, given the potential for vaccination to increase the risk of acquisition in large-cohort studies (11, 12). Interestingly, the observed level of protection (43%) in the M group closely matched that reported for women who were highly compliant with the dosing regime in CAPRISA 004 (5), although in our study with a limited number of animals, this did not reach statistical significance. Gel alone had no impact on viral kinetics; thus, at the systemic level, there was no evidence that the microbicide delayed or blunted infection. Indeed, while protection appeared to be an all-or-nothing event, the per-exposure probability of infection was reduced by 68% relative to that of the vaccine group, with a predicted per-exposure risk of infection of 0.068 relative to 0.087 for controls or 0.159 for the vaccine-only group (Table 2). Interestingly, there was no evidence of seroconversion in exposed but protected animals in the M group, despite repeated viral challenges (Fig. 5). These data are in accord with previous studies of humans and macaques where repeat vaginal exposure to 500 μg of recombinant gp140 failed to induce antibody responses (27, 28). The observed lack of seroconversion is in contrast to the reported seroconversion of a small number of subjects in the CAPRISA 004 trial (29) but is in accordance with the report on the Partners PrEP study (30). It is unclear if induced antibody responses in CAPRISA 004 were dependent upon limited replication that was insufficient to establish infection.

The V+M combination was the only group to show a statistically significant difference from the naive controls and the V-only group. Thus, any potential vaccine-related enhancement in the per-exposure risk of infection was mitigated by the V+M combination. Furthermore, there was a 63% risk reduction (OR, 0.39) in the V+M group relative to that of M alone, although this did not reach statistical significance. Nevertheless, the Kaplan-Meier curves from part 1 predict that 9.5 challenges would be required to infect 50% of the animals in group M, while too few animals (4/16) were infected in the V+M group to reliably calculate the same estimation for the combination group. Larger studies are needed to confirm or refute a difference between the V+M and M groups. However, the positive interaction between the microbicide and the vaccine appeared to have a sustained effect in part 2, where animals were challenged in the absence of a microbicide (interaction coefficient *P* = 0.13). This long-term benefit may indicate a durable effect of vaccination in this group or reflect the fact that repetitive challenges select animals with higher or increasing resistance to infection over time. The only observable impact of protected viral exposure on vaccine-induced immunity in the V+M group was an increased cellular response to Env relative to that of the V group after six repetitive challenges. The significance of this finding is unclear, as the level of cellular responses was not predictive of resistance to infection. Nevertheless, this echoes studies with the rhesus cytomegalovirus vector encoding SIV Gag, Rev-Tat-Nef, and Env that protected 50% of the animals from productive infection despite inducing equivalent cell-mediated immune responses in protected and unprotected animals (31). In contrast, antibody levels were too high to ascertain any boost effects of protected exposure in the V+M group. Irrespective of the mechanism, the persistent positive impact of the V+M combination is encouraging.

Previous studies of NHP have suggested a potential benefit of combining T-cell-based vaccine approaches with vaginal microbicides; however, sample sizes were small and the microbicide approaches used have yet to be tested in human efficacy studies (32, 33). This study is the first to assess the potential benefit of combining a microbicide with a humoral vaccine. We believe that our data suggest that a microbicide-vaccine combination might provide greater efficacy than either intervention alone. The observed benefits are likely to be improved with a vaccine that contains optimal B- and T-cell immunogens. Perhaps more importantly, these data indicate that provision of ARV prophylaxis ameliorates the potential for a vaccine-associated increased risk of infection. Although we assessed the efficacy of tenofovir gel, the protective effects would likely be similar or greater with oral prophylaxis, where compliance levels may be more reliable (34). This has important implications given that oral PrEP could be adopted as the baseline intervention for future HIV/AIDS vaccine trials. Our findings are contemporaneous with plans to evaluate a regimen similar to that of RV144, a partially effective vaccine thought to be mediated by humoral immunity in the absence of tier 2 neutralization, in South Africans (35, 36). This has important implications, given that oral PrEP could be adopted as the baseline intervention for future HIV/AIDS vaccine trials.
